# Perinatal mortality among term births: Informing decisions about singleton early term births in Western Australia

**DOI:** 10.1111/ppe.13124

**Published:** 2024-10-01

**Authors:** Ye’elah E. Berman, John P. Newnham, Elizabeth A. Nathan, Dorota A. Doherty, Kiarna Brown, Sarah V. Ward

**Affiliations:** ^1^ Medical School, Division of Obstetrics and Gynaecology University of Western Australia, King Edward Memorial Hospital Perth Western Australia Australia; ^2^ Royal Darwin Hospital Darwin Northern Territory Australia; ^3^ Menzies School of Health Research Darwin Northern Territory Australia

**Keywords:** gestational age, neonatal death, perinatal mortality, stillbirth, term births

## Abstract

**Background:**

To minimise the risk of perinatal mortality, clinicians and expectant mothers must understand the risks and benefits associated with continuing the pregnancy.

**Objectives:**

Report the gestation‐specific risk of perinatal mortality at term.

**Methods:**

Population‐based cohort study using linked health data to identify all singleton births at gestations 37–41 weeks, in Western Australia (WA) from 2009 to 2019. Lifetable analysis was used to combine the risk of each type of perinatal mortality and calculate the cumulative risk of perinatal mortality, termed the perinatal risk index (PRI). Rates of antepartum and intrapartum stillbirth and neonatal death, as well as the PRI, were examined for each gestational week at term by non‐Aboriginal and Aboriginal ethnicity. For non‐Aboriginal women, rates were also examined by time‐period (pre‐ vs. post‐WA Preterm Birth Prevention Initiative (the Initiative) rollout), primiparity, and obstetric risk.

**Results:**

There were 332,084 singleton term births, including 60 perinatal deaths to Aboriginal mothers (3.2 deaths per 1000 births to Aboriginal mothers) and 399 perinatal deaths to non‐Aboriginal mothers (1.3 deaths per 1000 births to non‐Aboriginal mothers). For non‐Aboriginal women, the PRI was at its lowest (PRI 0.80, 95% CI 0.61, 1.00) at 39 weeks gestation. For Aboriginal women, it was at its lowest at 38 weeks (PRI 2.43, 95% CI 0.48, 4.39) with similar risk at 39 weeks (PRI 2.68, 95% CI 1.22, 4.14). The PRI increased steadily after 39 weeks gestation. The risk of perinatal mortality was higher among Aboriginal women. The gestation‐specific perinatal mortality rates were similar by the time‐period, primiparity and obstetric risk.

**Conclusions:**

The gestational ages at term associated with the lowest risk of perinatal mortality reinforce that the recommendation not to deliver before 39 weeks without medical indication is applicable to both Aboriginal and non‐Aboriginal women giving birth in WA. There was no increase in the perinatal mortality rate associated with the introduction of the Initiative.

**Synopsis**

**Study question**

Which gestational week of birth at term carries the lowest risk of perinatal mortality?

**What is already known**

Timing of birth influences the risk of perinatal mortality. A previous study demonstrated that birth at 38 weeks was associated with the lowest cumulative risk of perinatal mortality.

**What the study adds**

Cumulative risk of perinatal mortality increased substantially after 39 weeks gestation, and there was a similar risk of death by time‐period (pre‐ vs. post‐Preterm Birth Prevention Initiative), primiparity and obstetric‐risk. Among non‐Aboriginal women the lowest risk of perinatal mortality was at 39 weeks. Among Aboriginal women the lowest risk was at 38 weeks, with similar risk at 39 weeks. These results support the policy that, without medical indication, waiting until 39 weeks for planned deliveries is safe among both population groups.

## 1 BACKGROUND

The Western Australian (WA) definition of perinatal mortality includes neonatal deaths, defined as deaths that occur within 28 days of a livebirth, and stillbirths occurring in foetuses greater than 20 weeks gestation or 400 g birthweight, and prior to (antepartum) or during (intrapartum) birth.^1^ Perinatal mortality affected 9.6 infants per 1000 in Australia in 2021, and was higher among women of Aboriginal and/or Torres Strait Islander ethnicity (hereafter respectfully referred to as Aboriginal), affecting 17.0 infants per 1000.^2^

Factors contributing to perinatal mortality in Australia include congenital anomalies, placental dysfunction, antepartum haemorrhage, perinatal infection, maternal age, residing in remote or socially disadvantaged areas, smoking during pregnancy, and pregnancies of twins or higher order multiples.^2^ Additionally, the timing of birth also influences the risk of perinatal mortality. Preterm deliveries (before 37 weeks) are associated with increased risk of neonatal morbidity^3^ and mortality,^4^ whereas the risk of stillbirth rises with increasing gestational age, particularly after 41 weeks.^5^ Recent research has revealed that compared to full term, birth at early term (37–38 weeks) is associated with increased risks of adverse outcomes in the neonatal period,^6^ childhood^7^, ^8^, ^9^, ^10^, ^11^, ^12^ and early adulthood.^13^ To reduce the burden of early birth, the WA Preterm Birth (PTB) Prevention Initiative (the Initiative) was rolled out across the state in 2014 with the aim of preventing PTB, as well as delaying early term births to 38 weeks or later when safe to do so. In practice, the advice was to delay to 39 weeks where safe, and this is how the policy has been referred to throughout the paper. The Initiative consisted of new clinical guidelines, outreach for clinicians, education for pregnant women and their families, and a new clinic for women at high risk of PTB.^14^ In the first year of the Initiative, the PTB rate was reduced across the state.^14^ After 5 years, the PTB rate continued to reduce at the established tertiary hospital among non‐Aboriginal women.^15^ With so many competing risks, the optimal timing of a planned birth to minimise the risk of mortality and morbidity remains uncertain, and due to these competing considerations, there is considerable variation in practice among obstetricians and midwives.^16^

To provide accurate counselling to expectant mothers on the optimal timing of birth, clinicians must weigh up the risks and benefits of continuing the pregnancy. This assessment requires an understanding of the risk of each type of perinatal mortality by gestational age, which varies depending on the denominator used.^17^, ^18^ While the crude perinatal mortality rate can be reported as the number of perinatal deaths divided by the number of births, the population at risk is different for each type of perinatal mortality, and the denominator in the probability equation must reflect this.^17^ To address these questions, Smith (2001)^18^ utilised lifetable analysis to examine the risk of death by antepartum stillbirth, intrapartum stillbirth, and neonatal death, and combined these risks by gestational age into a perinatal risk index (PRI).

In the current study we have applied these methods to birth data from 2009 to 2019, to determine which gestational age at term carries the lowest risk of perinatal mortality in WA. Application of the robust lifetable analysis method provides the opportunity to investigate the risk of all types of perinatal mortality combined, rather than reporting on stillbirth and neonatal death separately, as is typically done in Australian studies. Furthermore, our study is the first to report on deaths to 2019 on a WA cohort and is therefore uniquely positioned to assess associations between the perinatal mortality rate, and the delaying of unnecessary early births (as per the Initiative). To assess the impact of the Initiative on perinatal mortality, we investigated the perinatal mortality rate by time‐period (pre‐Initiative: 2009–2013 vs. Initiative: 2014–2019), and by individual year. Further analyses were conducted by non‐Aboriginal and Aboriginal ethnicity, and by primiparity. In the presence of particular maternal conditions or obstetric complications, early birth is the safest course of action,^19^ therefore, we also investigated the perinatal mortality rate by obstetric risk.

## 2 METHODS

### 2.1 Cohort selection

We conducted a population‐based cohort study using linked birth and death data from WA. The study population was obtained from the WA Midwives Notification System (MNS), which includes all live and stillbirths of at least 20 weeks gestation, or greater than 400 g birthweight. Singleton births from 37 to 41 weeks, between 1 January 2009 and 31 December 2019, were included in the study. Births from 42 weeks were excluded as extending pregnancy beyond this point is contrary to clinical recommendations, and the numbers were too small for the analysis. Multiples were excluded from the study as their mortality risk by gestational age differs from singletons. The cohort selection process and sample exclusions are shown in Figure S1. As the ethnicity of the father and baby was unknown, maternal Aboriginal status was obtained from the derived Aboriginal and Torres Strait Islander Flag dataset (derived flag) that is generated by the WA Data Linkage Unit.^20^ The derived flag, the WA Registry of Births, Deaths and Marriages (which includes all deaths in the state) and the Cause of Death Unit Record File were probabilistically linked to the MNS by the WA Data Linkage Unit.^21^, ^22^

### 2.2 Perinatal mortality outcomes

Stillbirths were recorded in the MNS as antepartum, intrapartum or stillbirths of unknown timing. Neonatal deaths, defined as deaths within 28 days of livebirth, were identified using the Registry of Births, Deaths and Marriages.

### 2.3 Exposure and risk classification

The primary exposure, gestational week at birth, and all other risk data were obtained from the MNS. Women were classified into either the pre‐Initiative (2009–2013), or the Initiative implementation (2014–2019) time‐period. They were classified as high obstetric risk if they had pre‐existing diabetes or hypertension, preeclampsia, gestational diabetes, antepartum haemorrhage, or pre‐labour rupture of membranes.

### 2.4 Statistical analyses

Participant characteristics were summarised using frequencies and proportions, by ethnicity and primiparity. Crude perinatal mortality and antepartum stillbirth rates were calculated by dividing the number of deaths at term, by the number of births at term. For crude intrapartum stillbirth rates, antepartum stillbirths were removed from the calculation, and for crude neonatal death rates, all stillbirths were removed from the calculation. Estimation of perinatal mortality was performed separately for non‐Aboriginal and Aboriginal women due to their different risk factors and rates.

Conditional rates and 95% confidence intervals (CI) of antepartum and intrapartum stillbirths, neonatal death, and overall cumulative risk of perinatal mortality (referred to as the PRI) were reported by week of gestation at term. Where the lower bound of a CI was negative, the value was replaced with zero. Details on these calculations are described in the paper that originally developed these methods,^18^ and in Table S1. For Aboriginal women, only overall death rates were reported as the numbers of birthing women were too small to allow for subgroup analyses. For non‐Aboriginal women, rates are reported overall and by time‐period, primiparity and obstetric risk.

Unadjusted and adjusted Poisson regression models for non‐Aboriginal women were used to evaluate whether the yearly rates of antepartum stillbirth, neonatal death, and all perinatal mortality differed from the reference year (2013, the last full year before the start of the Initiative). Modelling could not be performed for intrapartum stillbirths, or for Aboriginal women, as the number of events per year were too small. Maternal age, smoking during pregnancy, primiparity, caesarean at the last delivery, pre‐existing hypertension and diabetes, asthma, gestational diabetes, pre‐eclampsia, and other maternal conditions were available from the MNS and were considered for inclusion in regression models. The category ‘other maternal conditions’ included pre‐existing medical conditions diagnosed before the pregnancy, that may influence the pregnancy or pregnancy care.^23^ History of stillbirth and history of singleton PTB were calculated for each mother. Socioeconomic status was assigned based on the maternal residence postcode recorded on the MNS, using the Index of Relative Socio‐Economic Advantage and Disadvantage (IRSAD) score, derived by the Australian Bureau of Statistics.^24^ IRSAD scores were categorised into those in the lowest 20%, and those in the upper 80% of scores. Cause of death was obtained from the Cause of Death Unit Record file and coded using the International Statistical Classification of Diseases and Related Health Problems, 10th Revision (ICD‐10).^25^ Birth method (vaginal birth, instrumental vaginal birth, planned caesarean or emergency caesarean) was included for modelling of neonatal deaths. Counts less than five were supressed for privacy. Statistical software program SAS (version 9.4, Cary, NC: SAS Institute Inc.) was used for data analyses.

### 2.5 Missing data and sensitivity analysis

There were 67 stillbirths of unknown timing (antepartum/intrapartum) recorded (maximum: 17 in 2009, minimum: 1 in 2013). These stillbirths were excluded, with sensitivity analyses including these births firstly as antepartum stillbirths, and then as intrapartum stillbirths, conducted to confirm that the main results were not influenced by their exclusion. Poisson regression analyses were also run excluding deaths that had an underlying cause of ‘congenital malformations, deformations and chromosomal abnormalities’. IRSAD was missing for 2710 (0.8%) births and these were categorised as ‘unknown IRSAD’ for modelling. There were 45,663 (13.8%) births to multiparous women for whom a record of their previous pregnancy was not available. These women were classified as having an unknown history of PTB, and no prior stillbirth. All other data was complete.

### 2.6 Ethics approval

The study was approved by the Women and Newborn Health Service Human Research Ethics Committee (RGS0000002677), the Department of Health WA Human Research Ethics Committee (RGS0000000704) and the Western Australian Aboriginal Health Ethics Committee (965).

## 3 RESULTS

The characteristics of the study population, by ethnicity and primiparity, are shown in Table 1. There were 370,272 births during the study period, of which 335,710 were born at gestations from 37 to 41 weeks. After excluding 3559 twins or higher order multiples, and 67 stillbirths of unknown timing, the final sample size was 332,084. Of these births, 313,483 (94.4%) were to non‐Aboriginal mothers and 18,601 (5.6%) were to Aboriginal mothers. The perinatal mortality rate among Aboriginal mothers (60 deaths, 0.3%) was higher than that in non‐Aboriginal mothers (399 deaths, 0.1%).

**TABLE 1:** Characteristics of the study cohort – singleton births at term in Western Australia (2009–2019), by Aboriginal versus non‐Aboriginal ethnicity and primiparity.

	Non‐Aboriginal or Torres Strait Islander Women						Aboriginal or Torres Strait Islander Women					
	Overall		Primiparous		Multiparous		Overall		Primiparous		Multiparous	
	*N*	%	*N*	%	*N*	%	*N*	%	*N*	%	*N*	%
Maternal age >35 years	68,867	22.0	18,204	13.6	50,663	28.2	1486	8.0	101	1.7	1385	10.8
Any diabetes	26,593	8.5	11,382	8.5	15,211	8.5	1733	9.3	416	7.2	1317	10.3
Pre‐existing hypertension	2915	0.9	1149	0.9	1766	1.0	177	1.0	39	0.7	138	1.1
Pre‐eclampsia	4844	1.5	3282	2.5	1562	0.9	366	2.0	197	3.4	169	1.3
Antepartum haemorrhage	7296	2.3	3389	2.5	3907	2.2	341	1.8	107	1.9	234	1.8
Prelabour rupture of membranes	7171	2.3	4382	3.3	2789	1.6	526	2.8	219	3.8	307	2.4
Gestation at birth (weeks)												
37	26,633	8.5	10,651	8.0	15,982	8.9	2436	13.1	604	10.4	1832	14.3
38	81,135	25.9	28,068	21.0	53,067	29.5	4810	25.9	1283	22.2	3527	27.5
39	96,375	30.7	38,696	28.9	57,679	32.1	5515	29.6	1663	28.8	3852	30.0
40	78,200	24.9	38,590	28.8	39,610	22.0	4299	23.1	1550	26.8	2749	21.4
41	31,140	9.9	17,795	13.3	13,345	7.4	1541	8.3	680	11.8	861	6.7
Obstetric high‐risk^a^	46,307	14.8	22,281	16.7	24,026	13.4	2917	15.7	924	16.0	1993	15.5
Livebirths	313,084	99.9	133,618	99.9	179,466	99.9	18,541	99.7	5758	99.6	12,783	99.7
Antepartum stillbirth	206	0.1	103	0.1	103	0.1	31	0.2	14	0.2	17	0.1
Intrapartum stillbirth	31	0	14	0	17	0	5	0	<5	0	<5	0
Neonatal death	162	0.1	65	0	97	0.1	24	0.1	6	0.1	18	0.1
Perinatal mortality	399	0.1	182	0.1	217	0.1	60	0.3	22	0.4	38	0.3

*Note*: Exclusions: 67 singleton stillbirths of unspecified timing.

^a^ Obstetric high‐risk includes pre‐existing diabetes, pre‐existing hypertension, preeclampsia, gestational diabetes, antepartum haemorrhage for placenta praevia, placental abruption or other causes, and prelabour rupture of membranes.

The overall, gestation‐specific antepartum and intrapartum stillbirth rates, neonatal death rate, and the PRI for each week of gestation at term, by Aboriginality are shown in Table 2. For non‐Aboriginal women, the rate of antepartum stillbirth increased with each week of gestation. Birth at 39 weeks carried the lowest rate of both intrapartum stillbirth (0.03, 95% CI, 0.00, 0.15 per 1000) and neonatal death (0.27, 95% CI 0.17, 0.37 per 1000). The PRI was at its lowest (PRI 0.80, 95% CI 0.61, 1.00) at 39 weeks gestation, after which each additional week of gestation carried a higher risk of perinatal mortality than the week prior. Among Aboriginal women, the rate of antepartum stillbirth rose with advancing gestational age, and there were very few intrapartum stillbirths. The neonatal death rate decreased with advancing gestational age to a minimum at 40 weeks gestation (0.70, 95% CI, 0.00, 1.49 per 1000). The PRI was lowest at 38 weeks gestation (PRI 2.43, 95% CI 0.48, 4.39) which was similar to the risk at 39 weeks (PRI 2.68, 95% CI 1.22, 4.14). At 37 weeks and after 39 weeks, the risk of perinatal mortality was substantially increased. The PRI was higher at all gestational weeks among Aboriginal women than among non‐Aboriginal women (Figure S2A).

**TABLE 2:** Rates of antepartum stillbirth, intrapartum stillbirth, neonatal death and the PRI for singleton births at term in Western Australia (2009–2019), by week gestation and Aboriginal versus non‐Aboriginal ethnicity.

Gestation week	Non‐Aboriginal or Torres Strait Islander								Aboriginal or Torres Strait Islander							
	Antepartum stillbirth		Intrapartum stillbirth		Neonatal death		Perinatal risk index		Antepartum stillbirth		Intrapartum stillbirth		Neonatal death		Perinatal risk index	
	*N*	Rate^a^	*N*	Rate^b^	*N*	Rate^c^	*N*	Rate^d^	*N*	Rate^a^	*N*	Rate^b^	*N*	Rate^c^	*N*	Rate^d^
37	40	0.13 (0.09, 0.17)	<5	0.11 (0.00, 0.25)	31	1.17 (0.76, 1.58)	74	1.47 (0.71, 2.23)	6	0.34 (0.07, 0.62)	<5	0.41 (0.00, 1.15)	7	2.88 (0.75, 5.01)	14	4.08 (0.42, 7.75)
38	50	0.20 (0.15, 0.26)	10	0.12 (0.03, 0.22)	47	0.58 (0.41, 0.75)	107	0.94 (0.57, 1.3)	6	0.43 (0.09, 0.78)	<5	0.62 (0.04, 1.21)	6	1.25 (0.25, 2.25)	15	2.43 (0.48, 4.39)
39	53	0.33 (0.24, 0.42)	<5	0.03 (0.00, 0.15)	26	0.27 (0.17, 0.37)	82	0.80 (0.61, 1.00)	11	1.27 (0.52, 2.02)	<5	0.18 (0.00, 1.12)	6	1.09 (0.22, 1.96)	18	2.68 (1.22, 4.14)
40	49	0.69 (0.49, 0.88)	7	0.09 (0.00, 0.27)	36	0.46 (0.31, 0.61)	92	1.56 (1.31, 1.81)	6	1.60 (0.32, 2.88)	0	0.00 (0.00, 1.20)	<5	0.70 (0.00, 1.49)	9	3.54 (1.97, 5.11)
41	14	0.83 (0.40, 1.27)	8	0.26 (0.00, 0.58)	22	0.71 (0.41, 1.00)	44	2.73 (2.36, 3.10)	<5	2.41 (0.00, 5.73)	0	0.00 (0.00, 2.45)	<5	1.30 (0.00, 3.10)	<5	6.13 (3.8, 8.47)

^a^ Rate of antepartum stillbirth calculated as the number of antepartum stillbirths per gestational week, divided by the number of ongoing pregnancies, minus half of the births that occurred in that gestational week × 1000.

^b^ Rate of intrapartum stillbirth calculated as the number of intrapartum stillbirths per gestational week, divided by the number of deliveries at that gestational week, with antepartum stillbirths removed from the denominator × 1000.

^c^ Rate of neonatal death calculated as the number of neonatal deaths per gestational week, divided by the number of deliveries at that gestational week, with antepartum and intrapartum stillbirths removed from the denominator × 1000.

^d^ Cumulative probability of perinatal mortality calculated as 1‐the product of surviving antepartum stillbirth from gestational week 37 to birth, the probability of surviving without an intrapartum stillbirth in the given gestational week, and the probability of surviving neonatal death in the given gestational week. The PRI is the cumulative probability of perinatal mortality × 1000.

For non‐Aboriginal women, the rate estimates of mortality at each gestational week at term are also shown by subgroup. When subset by time‐period (Table 3), the PRI was similar but slightly lower for deliveries from 38 to 41 weeks during the Initiative period, compared to the pre‐Initiative time‐period (Figure S2B). During the pre‐Initiative time‐period, the PRI was at a minimum for deliveries at 38 weeks gestation (PRI 0.95, 95% CI 0.37, 1.53), and during the Initiative it was at a minimum at 39 weeks gestation (PRI 0.68, 95% CI 0.44, 0.92). The crude perinatal mortality rate was at a minimum in 2013 (0.93 deaths per 1000 births), and at a maximum in 2011 (1.98 deaths per 1000 births). Adjusted Poisson regression analysis showed that the risk of perinatal mortality was higher than the reference year (2013) in 2010 (aRR 2.02, 95% CI 1.26, 3.25), 2011 (aRR 2.18, 95% CI 1.37, 3.47), and 2018 (aRR 1.75, 95% CI 1.05, 2.90) (Table 4). Further investigation showed that the proportion of deaths with congenital anomalies coded as the cause of death was higher in 2009 (27.0%), 2014 (25.0%) and 2018 (26.3%) compared to only 11.1% of deaths in 2013. When births with ‘congenital malformations, deformations and chromosomal abnormalities’ listed as the cause of death were removed from the data, the perinatal mortality rate in 2018 was similar to the reference year (aRR 1.42, 95% CI 0.81, 2.48) (Table S2).

**TABLE 3:** Rates of antepartum stillbirth, intrapartum stillbirth, neonatal death and the PRI for singleton births at term in Western Australia, by week gestation and time‐period, in non‐Aboriginal or Torres Strait Islander women.

Gestation week	Antepartum stillbirth				Intrapartum stillbirth				Neonatal death				Perinatal risk index			
	2009–2013^a^		2014–2019^b^		2009–2013^a^		2014–2019^b^		2009–2013^a^		2014–2019^b^		2009–2013^a^		2014–2019^b^	
	*N*	Rate^c^	*N*	Rate^c^	*N*	Rate^d^	*N*	Rate^d^	*N*	Rate^e^	*N*	Rate^e^	*N*	Rate^f^	*N*	Rate^f^
37	25	0.19 (0.11, 0.26)	15	0.09 (0.04, 0.13)	<5	0.19 (0.00, 0.45)	<5	0.06 (0.00, 0.21)	11	1.03 (0.42, 1.63)	20	1.26 (0.71, 1.81)	38	1.43 (0.28, 2.57)	36	1.50 (0.49, 2.51)
38	27	0.24 (0.15, 0.34)	23	0.17 (0.10, 0.24)	5	0.15 (0.00, 0.31)	5	0.11 (0.00, 0.22)	17	0.49 (0.26, 0.73)	30	0.64 (0.41, 0.87)	49	0.95 (0.37, 1.53)	58	0.92 (0.45, 1.40)
39	23	0.31 (0.19, 0.44)	30	0.35 (0.23, 0.48)	<5	0.03 (0.00, 0.20)	<5	0.04 (0.00, 0.19)	14	0.35 (0.17, 0.54)	12	0.21 (0.09, 0.33)	38	0.96 (0.64, 1.29)	44	0.68 (0.44, 0.92)
40	25	0.72 (0.44, 1.00)	24	0.65 (0.39, 0.91)	<5	0.11 (0.00, 0.38)	<5	0.07 (0.00, 0.32)	19	0.51 (0.28, 0.74)	17	0.42 (0.22, 0.62)	48	1.72 (1.31, 2.12)	44	1.43 (1.11, 1.74)
41	8	0.95 (0.29, 1.62)	6	0.71 (0.14, 1.28)	<5	0.13 (0.00, 0.62)	6	0.38 (0.00, 0.80)	15	0.98 (0.48, 1.47)	7	0.44 (0.12, 0.77)	25	3.04 (2.49, 3.60)	19	2.44 (1.96, 2.92)

^a^ Pre‐ WA PTB Prevention Initiative: 2009–2013.

^b^ WA PTB Prevention Initiative: 2014–2019.

^c^ Rate of antepartum stillbirth calculated as the number of antepartum stillbirths per gestational week, divided by the number of ongoing pregnancies, minus half of the births that occurred in that gestational week × 1000.

^d^ Rate of intrapartum stillbirth calculated as the number of intrapartum stillbirths per gestational week, divided by the number of deliveries at that gestational week, with antepartum stillbirths removed from the denominator × 1000.

^e^ Rate of neonatal death calculated as the number of neonatal deaths per gestational week, divided by the number of deliveries at that gestational week, with antepartum and intrapartum stillbirths removed from the denominator × 1000.

^f^ Cumulative probability of perinatal mortality calculated as 1‐the product of surviving antepartum stillbirth from gestational week 37 to birth, the probability of surviving without an intrapartum stillbirth in the given gestational week, and the probability of surviving neonatal death in the given gestational week. The PRI is the cumulative probability of perinatal mortality × 1000.

**TABLE 4:** Rates and rate ratios for antepartum stillbirth, intrapartum stillbirth, neonatal death and the PRI for singleton births at term in Western Australia, by year, in non‐Aboriginal or Torres Strait Islander women (2009–2019).

Year	Antepartum stillbirth				Intrapartum stillbirth		Neonatal death				Perinatal mortality			
	*N*	Rate	Unadjusted RR	Adjusted RR	*N*	Rate	*N*	Rate	Unadjusted RR	Adjusted RR	*N*	Rate	Unadjusted RR (95% CI)	Adjusted RR
		(per 1000)	(95% CI)	(95% CI)		(per 1000)		(per 1000)	(95% CI)	(95% CI)		(per 1000)		(95% CI)
2009	15	0.57	1.11	1.16	<5	0.04	21	0.80	2.33	2.32	37	1.41	1.52	1.56
			(0.54, 2.27)	(0.57, 2.40)					(1.10, 4.95)	(1.09, 4.98)			(0.93, 2.50)	(0.95, 2.57)
2010	27	1.02	1.99	2.07	<5	0.08	19	0.72	2.10	2.13	48	1.82	1.96	2.02
			(1.06, 3.73)	(1.10, 3.92)					(0.98, 4.51)	(0.98, 4.60)			(1.23, 3.14)	(1.26, 3.25)
2011	32	1.17	2.28	2.35	7	0.26	15	0.55	1.60	1.61	54	1.98	2.14	2.18
			(1.23, 4.21)	(1.27, 4.35)					(0.72, 3.57)	(0.72, 3.6)			(1.35, 3.39)	(1.37, 3.47)
2012	19	0.66	1.28	1.29	<5	0.07	11	0.38	1.12	1.11	32	1.11	1.20	1.21
			(0.65, 2.53)	(0.66, 2.55)					(0.47, 2.63)	(0.47, 2.62)			(0.72, 2.00)	(0.72, 2.01)
2013	15	0.51	1.00 (Reference)	1.00 (Reference)	<5	0.07	10	0.34	1.00 (Reference)	1.00 (Reference)	27	0.93	1.00 (Reference)	1.00 (Reference)
2014	14	0.47	0.91	0.90	<5	0.13	14	0.47	1.37	1.37	32	1.07	1.16	1.16
			(0.44, 1.89)	(0.44, 1.87)					(0.61, 3.08)	(0.61, 3.09)			(0.69, 1.93)	(0.69, 1.93)
2015	18	0.60	1.17	1.17	<5	0.10	16	0.54	1.56	1.58	37	1.24	1.34	1.35
			(0.59, 2.33)	(0.59, 2.32)					(0.71, 3.45)	(0.72, 3.49)			(0.82, 2.20)	(0.82, 2.22)
2016	18	0.59	1.15	1.14	<5	0.10	12	0.39	1.15	1.15	33	1.08	1.17	1.17
			(0.58, 2.28)	(0.58, 2.26)					(0.50, 2.66)	(0.50, 2.65)			(0.70, 1.94)	(0.71, 1.95)
2017	18	0.62	1.20	1.32	<5	0.07	12	0.41	1.20	1.27	32	1.10	1.19	1.29
			(0.61, 2.39)	(0.66, 2.64)					(0.52, 2.78)	(0.55, 2.95)			(0.71, 1.98)	(0.77, 2.16)
2018	16	0.57	1.10	1.36	<5	0.14	18	0.64	1.86	2.13	38	1.34	1.45	1.75
			(0.54, 2.22)	(0.66, 2.81)					(0.86, 4.02)	(0.96, 4.76)			(0.89, 2.38)	(1.05, 2.9)
2019	14	0.50	0.97	1.19	<5	0.04	14	0.50	1.45	1.67	29	1.03	1.11	1.34
			(0.47, 2.00)	(0.57, 2.52)					(0.64, 3.27)	(0.72, 3.87)			(0.66, 1.88)	(0.79, 2.29)

*Note*: Antepartum stillbirths were removed from the intrapartum stillbirth rates. All stillbirths have been removed from the neonatal death rates. Modelling was not conducted for intrapartum stillbirths because the numbers each year were too small. Unadjusted numbers and rates are still provided. Variables considered for inclusion in adjusted Poisson regression models were primiparity, smoking during pregnancy, other maternal conditions, history of preterm birth, history of stillbirth, IRSAD in the lowest 20%, maternal age, pre‐existing hypertension, pre‐existing diabetes, gestational diabetes, pre‐eclampsia, asthma, caesarean at last delivery. Neonatal death was additionally adjusted for method of birth (vaginal, vaginal instrumental, planned caesarean, emergency caesarean).

When categorised by primiparity (Table 5), the PRI (Figure S2C) was at its minimum at 39 weeks gestation for both primiparous (PRI 0.74, 95% CI 0.42, 1.06) and multiparous (PRI 0.83, 95% CI 0.59, 1.08) women. When subset by obstetric risk (Table 6), the PRI (Figure S2D) was lowest at 39 weeks for low‐risk women (PRI 0.76, 95% CI 0.55, 0.98) and at 38 weeks for high‐risk women (PRI 0.87, 95% CI 0.23, 1.52). For both categorisations, the PRI was similar at all term gestations.

**TABLE 5:** Rates of antepartum stillbirth, intrapartum stillbirth, neonatal death and the PRI for singleton births at term in Western Australia (2009–2019), by week gestation and primiparity in non‐Aboriginal or Torres Strait Islander women.

Gestation week	Antepartum stillbirth				Intrapartum stillbirth				Neonatal death				Perinatal risk index			
	Primiparous		Multiparous		Primiparous		Multiparous		Primiparous		Multiparous		Primiparous		Multiparous	
	*N*	Rate^a^	*N*	Rate^a^	*N*	Rate^b^	*N*	Rate^b^	*N*	Rate^c^	*N*	Rate^c^	*N*	Rate^d^	*N*	Rate^d^
37	17	0.13 (0.07, 0.19)	23	0.13 (0.08, 0.19)	<5	0.19 (0.00, 0.41)	<5	0.06 (0.00, 0.24)	9	0.85 (0.29, 1.4)	22	1.38 (0.80, 1.95)	28	1.22 (0.19, 2.26)	46	1.64 (0.57, 2.71)
38	23	0.21 (0.12, 0.30)	27	0.20 (0.12, 0.27)	<5	0.14 (0.00, 0.31)	6	0.11 (0.00, 0.23)	21	0.75 (0.43, 1.07)	26	0.49 (0.30, 0.68)	48	1.13 (0.49, 1.76)	59	0.83 (0.39, 1.28)
39	30	0.39 (0.25, 0.53)	23	0.28 (0.17, 0.39)	0	0.00 (0.00, 0.20)	<5	0.05 (0.00, 0.19)	8	0.21 (0.06, 0.35)	18	0.31 (0.17, 0.46)	38	0.74 (0.42, 1.06)	44	0.83 (0.59, 1.08)
40	23	0.61 (0.36, 0.86)	26	0.77 (0.47, 1.07)	<5	0.10 (0.00, 0.35)	<5	0.08 (0.00, 0.35)	15	0.39 (0.19, 0.59)	21	0.53 (0.30, 0.76)	42	1.53 (1.14, 1.92)	50	1.60 (1.27, 1.93)
41	10	1.04 (0.40, 1.69)	<5	0.55 (0.01, 1.09)	<5	0.22 (0.00, 0.70)	<5	0.30 (0.00, 0.70)	12	0.67 (0.29, 1.06)	10	0.75 (0.29, 1.21)	26	2.76 (2.24, 3.28)	18	2.70 (2.19, 3.21)

^a^ Rate of antepartum stillbirth calculated as the number of antepartum stillbirths per gestational week, divided by the number of ongoing pregnancies, minus half of the births that occurred in that gestational week × 1000.

^b^ Rate of intrapartum stillbirth calculated as the number of intrapartum stillbirths per gestational week, divided by the number of deliveries at that gestational week, with antepartum stillbirths removed from the denominator × 1000.

^c^ Rate of neonatal death calculated as the number of neonatal deaths per gestational week, divided by the number of deliveries at that gestational week, with antepartum and intrapartum stillbirths removed from the denominator × 1000.

^d^ Cumulative probability of perinatal mortality calculated as 1‐the product of surviving antepartum stillbirth from gestational week 37 to birth, the probability of surviving without an intrapartum stillbirth in the given gestational week, and the probability of surviving neonatal death in the given gestational week. The PRI is the cumulative probability of perinatal mortality × 1000.

**TABLE 6:** Rates of antepartum stillbirth, intrapartum stillbirth, neonatal death and the PRI for singleton births at term in Western Australia (2009–2019), by week gestation and low and high obstetric risk^a^ in non‐Aboriginal or Torres Strait Islander women.

Gestation week	Antepartum stillbirth				Intrapartum stillbirth				Neonatal death				Perinatal risk index			
	Low		High		Low		High		Low		High		Low		High	
	N	Rate^b^	N	Rate^b^	N	Rate^c^	N	Rate^c^	N	Rate^d^	N	Rate^d^	N	Rate^e^	N	Rate^e^
37	33	0.13 (0.08, 0.17)	7	0.17 (0.04, 0.29)	<5	0.11 (0.00, 0.27)	<5	0.13 (0.00, 0.41)	21	1.13 (0.65, 1.61)	10	1.25 (0.48, 2.03)	56	1.42 (0.44, 2.40)	18	1.63 (0.40, 2.85)
38	44	0.20 (0.14, 0.26)	6	0.20 (0.04, 0.36)	8	0.12 (0.01, 0.23)	<5	0.12 (0.00, 0.34)	39	0.60 (0.41, 0.79)	8	0.49 (0.15, 0.83)	91	0.95 (0.51, 1.40)	16	0.87 (0.23, 1.52)
39	49	0.34 (0.25, 0.44)	<5	0.26 (0.01, 0.51)	<5	0.01 (0.00, 0.14)	<5	0.15 (0.00, 0.43)	21	0.25 (0.14, 0.36)	5	0.38 (0.05, 0.72)	71	0.76 (0.55, 0.98)	11	1.03 (0.54, 1.52)
40	45	0.68 (0.48, 0.88)	<5	0.77 (0.02, 1.53)	7	0.10 (0.00, 0.29)	0	0.00 (0.00, 0.63)	34	0.48 (0.32, 0.64)	<5	0.27 (0.00, 0.64)	86	1.59 (1.32, 1.86)	6	1.28 (0.66, 1.89)
41	13	0.81 (0.37, 1.25)	<5	1.31 (0.00, 3.88)	7	0.24 (0.00, 0.56)	<5	0.73 (0.00, 2.64)	22	0.74 (0.43, 1.05)	0	0.00 (0.00, 0.00)	42	2.73 (2.34, 3.11)	<5	2.77 (1.58, 3.97)

^a^ Women were classified as high obstetric risk if they had pre‐existing diabetes or hypertension, preeclampsia, gestational diabetes, antepartum haemorrhage, or pre‐labour rupture of membranes.

^b^ Rate of antepartum stillbirth calculated as the number of antepartum stillbirths per gestational week, divided by the number of ongoing pregnancies, minus half of the births that occurred in that gestational week × 1000.

^c^ Rate of intrapartum stillbirth calculated as the number of intrapartum stillbirths per gestational week, divided by the number of deliveries at that gestational week, with antepartum stillbirths removed from the denominator × 1000.

^d^ Rate of neonatal death calculated as the number of neonatal deaths per gestational week, divided by the number of deliveries at that gestational week, with antepartum and intrapartum stillbirths removed from the denominator × 1000.

^e^ Cumulative probability of perinatal mortality calculated as 1‐the product of surviving antepartum stillbirth from gestational week 37 to birth, the probability of surviving without an intrapartum stillbirth in the given gestational week, and the probability of surviving neonatal death in the given gestational week. The PRI is the cumulative probability of perinatal mortality × 1000.

## 4 COMMENT

### 4.1 Principal findings

Our study used lifetable analysis to examine the gestation‐specific risk of perinatal mortality at term by ethnicity, time‐period, primiparity, and obstetric risk. Among non‐Aboriginal women, birth at 39 weeks carried the lowest risk of perinatal mortality while among Aboriginal women, the lowest risk was at 38 weeks, with a similar risk at 39 weeks. The risk of perinatal mortality was higher for Aboriginal women compared to non‐Aboriginal women at all term gestations. The gestation‐specific PRI was similar between the pre‐Initiative and Initiative time periods, confirming that the Initiative was not associated with an increase in the perinatal mortality rate. No substantial differences were observed in the risk of perinatal mortality by primiparity, or obstetric risk.

### 4.2 Strengths of the study

Our study population included 11 years of birth data for the whole of WA. This extensive sample size allowed us to conduct subgroup analyses, and the use of a population‐based cohort study design minimised systematic error. These are the most recently reported perinatal mortality data for WA and allowed us to evaluate, for the first time, whether the introduction of the Initiative was associated with a change in the perinatal mortality rate. The availability of ethnicity data enabled separate analyses in Aboriginal women, adding valuable knowledge on a group of women with a relatively higher rate of perinatal mortality. Undertaking lifetable analysis allowed us to use appropriate denominators for each type of perinatal mortality, while still producing an overall gestation‐specific risk of death. To the best of our knowledge, this technique has not previously been applied to Australian data.

### 4.3 Limitations of the data

Despite our large sample size, the low rate of perinatal mortality limited our ability to detect differences between groups and did not allow for subgroup analysis among Aboriginal women. Furthermore, it would have been preferable to exclude babies born with congenital anomalies incompatible with life from analyses. While we could identify babies who died after birth due to ‘congenital malformations, deformations and chromosomal abnormalities’, we were not able to identify babies with congenital anomalies who lived past 28 days, or who were stillborn. However, research in WA has shown that in recent years, deaths due to congenital anomalies incompatible with life have increasingly occurred as stillbirths at preterm gestations^23^ and consequently, these births would not have been included in our analyses as we investigated perinatal mortality from 37 to 41 weeks. Our findings cannot be generalised to post term pregnancies as births after 41 weeks were excluded.

### 4.4 Interpretation

Our study found a higher rate of perinatal mortality among Aboriginal mothers compared to non‐Aboriginal mothers. This is consistent with previous literature showing that despite an observed reduction in stillbirths and neonatal deaths in both populations, the relatively higher mortality rate among Aboriginal babies has remained the same over the last 35 years.^26^ Previous research showed that the modifiable risk factors of smoking, alcohol, drug misuse and assault can account for 20% of perinatal mortality among Aboriginal women giving birth in WA.^27^ The higher burden of maternal health conditions among Aboriginal women^15^ likely also contributes to their higher burden of perinatal mortality. While in our cohort the proportion of women classified as obstetric high‐risk was only slightly higher among Aboriginal women than among non‐Aboriginal women, the difference was three times larger when the cohort was not limited to term births. This indicates that Aboriginal women with obstetric complications are being delivered earlier than non‐Aboriginal women with obstetric complications. While some initiatives have shown promise in reducing these risk factors for Aboriginal women,^28^, ^29^ they are particularly difficult to modify due to the economic and social disadvantage they face resulting from colonisation.^30^

In our study, birth at 39 weeks carried the lowest perinatal mortality rate for non‐Aboriginal women. While we found that birth at 38 weeks carried the lowest risk for Aboriginal women, there was very little difference in risk to birth at 39 weeks, implying that the optimal timing of birth does not vary by Aboriginal versus non‐Aboriginal ethnicity. It could therefore be concluded that in the absence of medical indication, it is similarly safe for births to be planned for 38 or 39 weeks. However, due to the adverse^6^, ^7^, ^8^, ^13^ health and developmental outcomes for babies born at early term, 39 weeks is considered the preferred gestation.

As the strategies of the Initiative, were designed with the aim of safely delaying early birth,^14^ it is vital to verify that there was no associated increase in perinatal mortality. Our study found a similar risk of perinatal mortality by gestational age at term between the pre‐Initiative and Initiative time periods, confirming the Initiative was not associated with an increase in perinatal mortality. While adjusted Poisson regression analysis showed an increase in the perinatal mortality rate in 2018, compared to 2013 (the last year before the Initiative), the difference was small, and the crude perinatal mortality rate in 2018 was lower than most pre‐Initiative years. Furthermore, when deaths coded in the Cause of Death data as congenital anomalies were excluded, the rate in 2018 was similar to the reference year. The higher perinatal mortality rate in 2010 and 2011 (pre‐Initiative), was driven by an abnormally high antepartum stillbirth rate. Investigations^31^, ^32^ have not revealed the cause of this increase.

The PRI was similar for primiparous and multiparous women. This result is inconsistent with previous research which has shown that primiparous women are at higher risk of stillbirth and neonatal death at term gestations.^16^, ^33^ The differing results could be due to inter‐country differences between our study and the aforementioned research – both in the composition of the birthing population, and in clinical practice. Likewise, there were no discernible differences in the PRI by gestation at term between women who were classified as high or low obstetric risk, which seems counter intuitive, considering the conditions used to classify women as high obstetric risk are associated with perinatal mortality. This result is likely a reflection of appropriately timed obstetric intervention preventing perinatal mortality among these high‐risk women, with 23.3% of obstetric high‐risk women delivering prior to 37 weeks, compared to only 5.4% of obstetric low‐risk women.

Our findings have important implications for clinical practice in reducing the burden of perinatal mortality. While the lowest risk of perinatal mortality was observed at 39 weeks gestation for non‐Aboriginal women, and 38 weeks for Aboriginal women, our calculations were based on a combination of spontaneous and iatrogenic deliveries, and therefore it does not follow that women should then automatically have their babies delivered at these gestations. Such a policy may increase the rate of obstetric intervention to unacceptable levels. As only 5.6% of births in WA were to Aboriginal mothers, the number of deaths among this group was small and sub‐group analyses could not be performed for this population. It would be valuable to repeat this analysis with additional years of data or including data from another state, to increase the sample size and confirm that our conclusions hold. While the use of lifetable analysis was a strength of this study, alternative statistical methods allowing for smaller sample sizes could also be considered in future research.

## 5 CONCLUSIONS

Birth at 39 weeks gestation was associated with the lowest risk of perinatal mortality for non‐Aboriginal women, while birth at 38 weeks was associated with the lowest risk for Aboriginal women, with the risk at 39 weeks being similar. These results indicate that the optimal timing of birth at term to reduce perinatal mortality does not vary by Aboriginal versus non‐Aboriginal ethnicity. While our sample of Aboriginal women was relatively small, our results support the Initiative policy that for both ethnicities, it is safe from a perinatal mortality perspective, not to plan birth prior to 39 weeks in the absence of medical or obstetric reasons for earlier birth. This was further demonstrated by the similar rates of perinatal mortality between the pre‐Initiative, and Initiative time‐periods. The use of lifetable analysis to combine the different types of perinatal mortality using appropriate denominators, provides more accurate data on the risk of perinatal mortality at term by week gestation, and may assist clinicians in supporting women in their decisions about when is the safest time to plan birth.

## AUTHOR CONTRIBUTIONS

Y.E.B. analysed and interpreted the data and drafted the manuscript. J.P.N. conceived of the project, designed the work, interpreted the data and edited and revised the manuscript. E.A.N. analysed the data and edited and revised the manuscript. D.A.D. conceived of the project, designed the work, analysed and interpreted the data and edited and revised the manuscript. K.B. designed the work, interpreted the data and edited and revised the manuscript. S.V.W. designed the work, interpreted the data and edited and revised the manuscript.

## FUNDING INFORMATION

This study was supported by the National Health and Medical Research Council of Australia (Partnership Grant APP1151853 www.nhmrc.gov.au), the Women and Infants Research Foundation of Western Australia (www.wirf.com.au), Channel 7 Telethon (www.Telethon7.com), the Commonwealth Department of Health and Ageing, and the McCusker Foundation. None of the study sponsors had any role in the design, data collection, analysis, interpretation of data, writing of the manuscript or decision to submit the paper for publication.

## CONFLICT OF INTEREST STATEMENT

The authors declare that they have no competing interests.

## PATIENT CONSENT STATEMENT

A waiver of consent was obtained for this study.

## PERMISSION TO REPRODUCE MATERIAL FROM OTHER SOURCES

This article does not make use of previously published material.

## Supporting information


Figure S1

Figure S2

Tables S1–S2

## Data Availability

The authors do not have permission to share patient‐level data extracted from the Data Linkage Unit of the Department of Health of Western Australia. Data can only be made available to researchers who apply to the Department of Health of Western Australia's Human Research Ethics Committee (https://ww2.health.wa.gov.au/Articles/A_E/Department‐of‐Health‐Human‐Research‐Ethics‐Committee) and Data Linkage Unit (www. datalinkagewa.org.au). Please contact the lead author, Ye'elah Berman (yeelah.berman@uwa.edu.au) to discuss the availability of data further.
