# Supplementary material for: Perinatal mortality among term births: Informing decisions about singleton early term births in Western Australia
Source: Paediatr Perinat Epidemiol. 2024 Oct 1;38(8):717–29. doi: 10.1111/ppe.13124 (PMC11603756; doi:10.1111/ppe.13124)
Supplement: Supplementary file 1 — Figure S1 [file PPE-38-717-s001.pptx]

## Slide 1
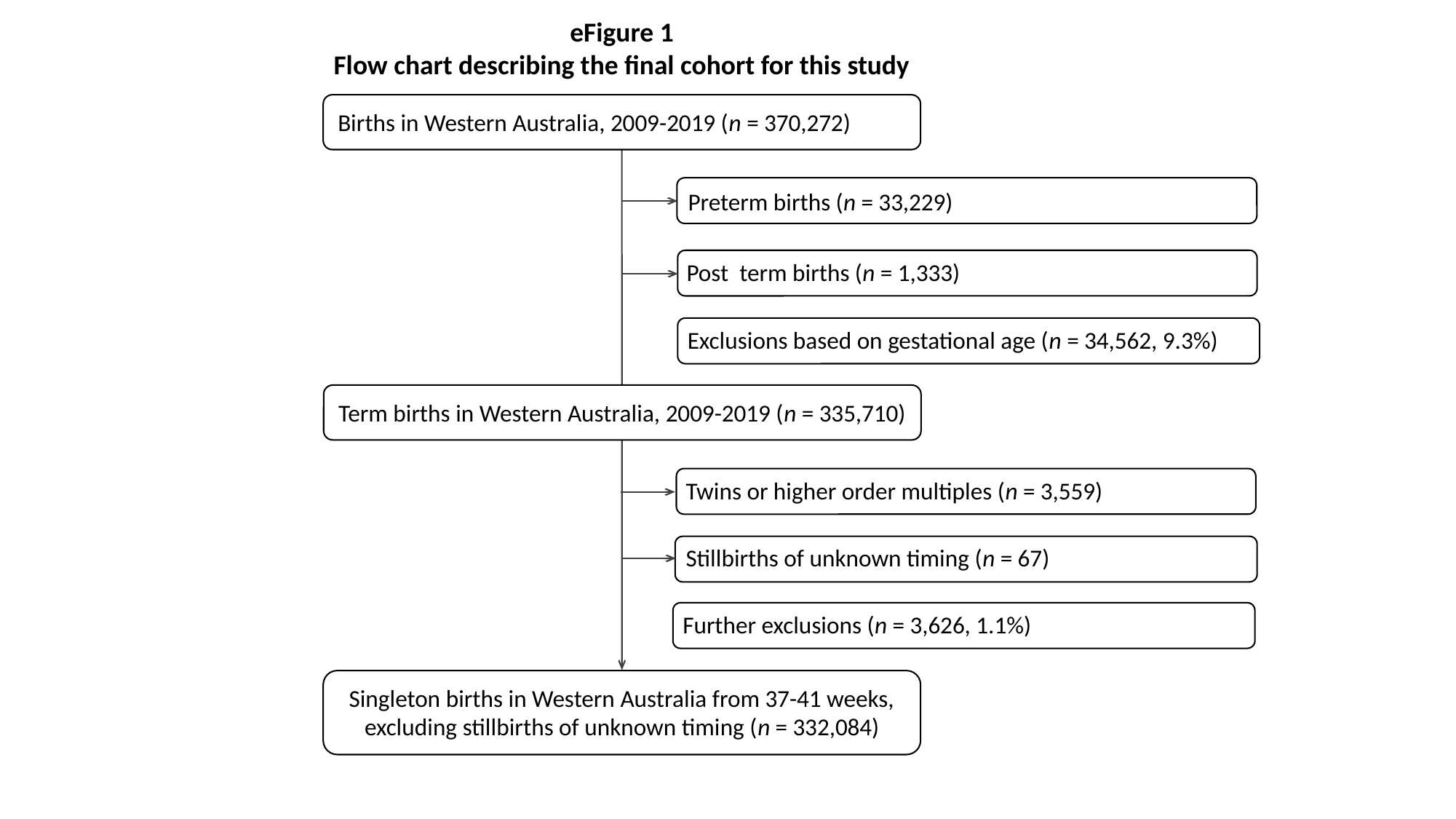

eFigure 1
Flow chart describing the final cohort for this study
Births in Western Australia, 2009-2019 (n = 370,272)
Preterm births (n = 33,229)
Post term births (n = 1,333)
Exclusions based on gestational age (n = 34,562, 9.3%)
Singleton births in Western Australia from 37-41 weeks, excluding stillbirths of unknown timing (n = 332,084)
Term births in Western Australia, 2009-2019 (n = 335,710)
Twins or higher order multiples (n = 3,559)
Stillbirths of unknown timing (n = 67)
Further exclusions (n = 3,626, 1.1%)
