# Supplementary material for: Perinatal mortality among term births: Informing decisions about singleton early term births in Western Australia
Source: Paediatr Perinat Epidemiol. 2024 Oct 1;38(8):717–29. doi: 10.1111/ppe.13124 (PMC11603756; doi:10.1111/ppe.13124)
Supplement: Supplementary file 2 — Figure S2 [file PPE-38-717-s003.pdf]

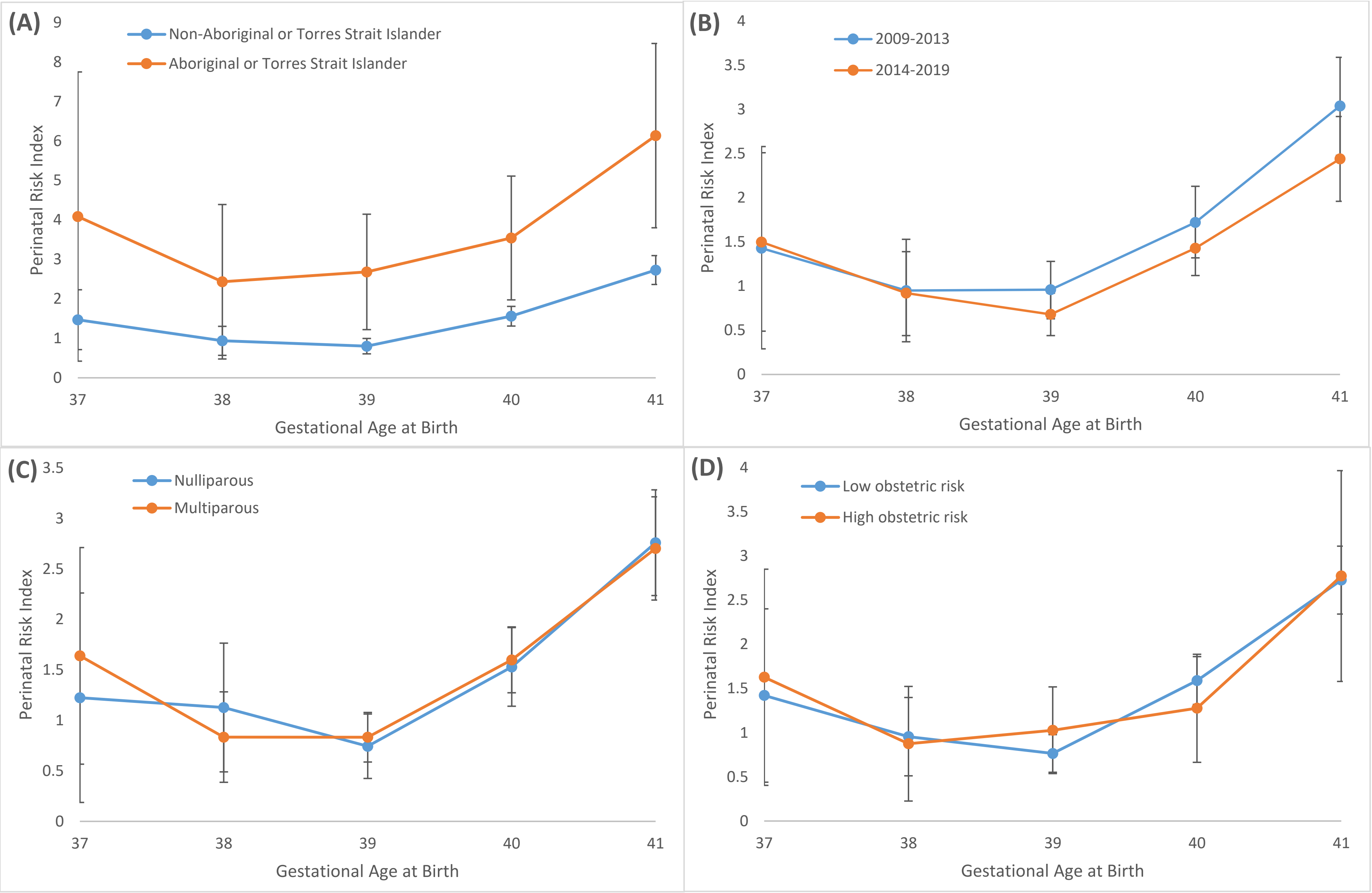

eFigure 1. Perinatal Risk Index by gestational week at delivery for singleton births at term in Western Australia (2009-2019), by (A) Aboriginal or Torres Strait Islander versus non-Aboriginal or Torres Strait Islander, (B) Pre-WA PTB Prevention Initiative: 2009-2013 versus WA PTB Prevention Initiative: 2014-2019, non-Aboriginal or Torres Strait Islander women, (C) Nulliparous versus multiparous, non-Aboriginal or Torres Strait Islander women, and (D) Low obstetric risk versus high obstetric risk, non-Aboriginal or Torres Strait Islander women.
