# Supplementary material for: Perinatal mortality among term births: Informing decisions about singleton early term births in Western Australia
Source: Paediatr Perinat Epidemiol. 2024 Oct 1;38(8):717–29. doi: 10.1111/ppe.13124 (PMC11603756; doi:10.1111/ppe.13124)
Supplement: Supplementary file 3 — Tables S1–S2 [file PPE-38-717-s002.docx]

**eTable 1. Calculation methods for the antepartum stillbirth, intrapartum stillbirth, neonatal death rate and the Perinatal Risk Index (PRI) for lifetable analysis.**

| **Antepartum Stillbirth** | As antepartum stillbirths are related to ongoing pregnancies, the gestation-specific antepartum stillbirth rate was calculated as the number of antepartum stillbirths in that week, divided by the number of ongoing pregnancies, minus half of the births that occurred in that gestational week.  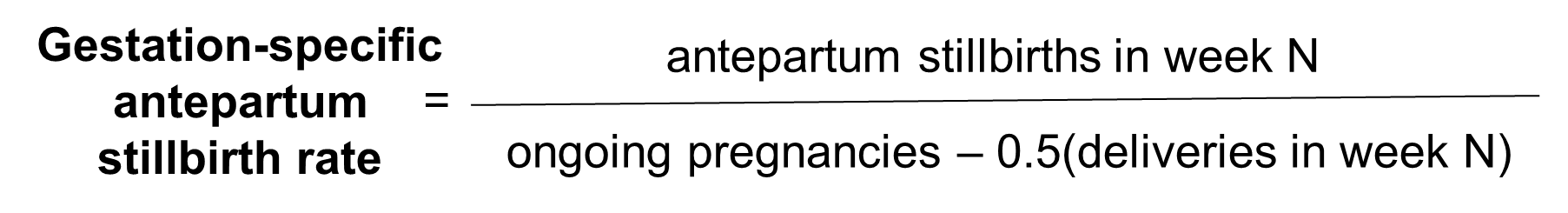  The cumulative probability of death from an antepartum stillbirth was calculated as 1 – the probability of antepartum stillbirth from 37 weeks gestation, and was only used for calculation of the PRI.  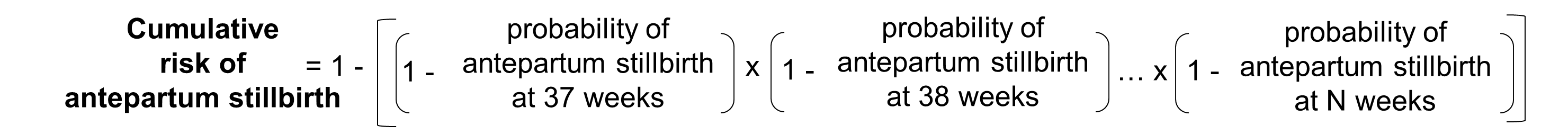 |
| --- | --- |
| **Intrapartum Stillbirth** | As intrapartum stillbirths relate to all births in a gestational week, with the exception of antepartum stillbirths, the gestation-specific intrapartum stillbirth rate was calculated as the number of intrapartum stillbirths at that gestation, divided by the number of deliveries at that gestational week, with antepartum stillbirths removed from the denominator.  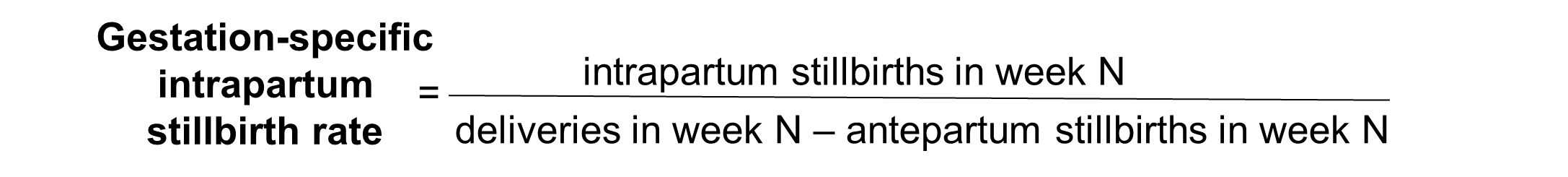 |
| **Neonatal Death** | As neonatal deaths relate to all live births in a gestational week, the gestation-specific neonatal death rate was calculated as the number of neonatal deaths at that gestation, divided by the number of deliveries at that gestational week, with antepartum and intrapartum stillbirths removed from the denominator.  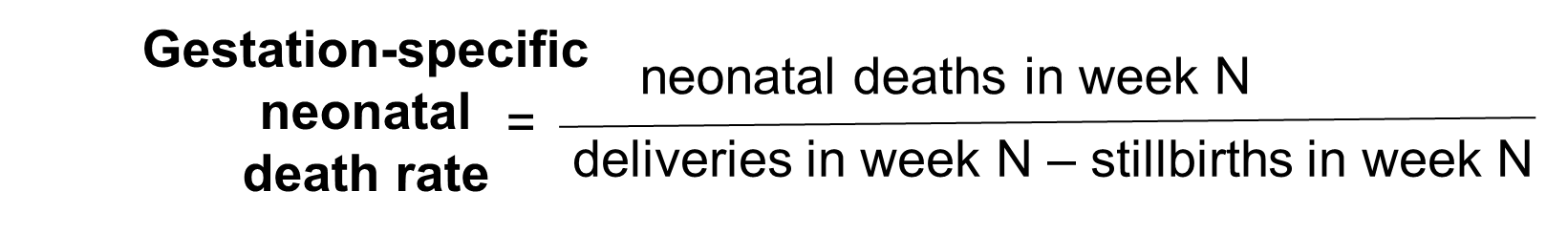 |
| **Perinatal Risk Index** | The gestation-specific cumulative probability of perinatal mortality was calculated as 1-the product of surviving antepartum stillbirth from gestational week 37 to birth, the probability of surviving without an intrapartum stillbirth in the given gestational week, and the probability of surviving neonatal death in the given gestational week. The PRI is the cumulative probability of perinatal mortality x 1000.  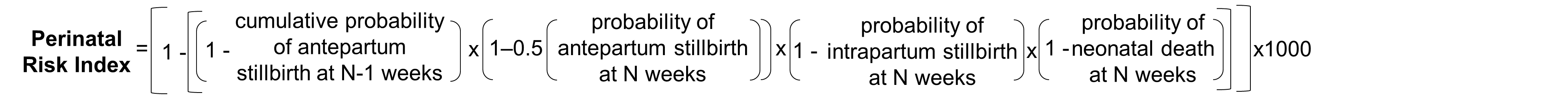 |

**eTable 2. Rates and rate ratios of perinatal mortality for singleton births at term in Western Australia, by year, in non-Aboriginal or Torres Strait Islander women (2009-2019), with deaths due to congenital malformations, deformations and chromosomal abnormalities excluded.**

|  | **Perinatal mortality** | | | | |
| --- | --- | --- | --- | --- | --- |
| **Year** | **N deaths excluded^a^ (%)** | **N deaths remaining** | **Rate** | **Unadjusted RR  (95% CI)** | **Adjusted RR** |
|  |  |  | **(per 1000)** |  | **(95% CI)** |
| **2009** | 10 (27.0) | 27 | 1.030 | 1.25 | 1.27 |
|  |  |  |  | (0.72, 2.16) | (0.73, 2.21) |
| **2010** | 5 (10.4) | 43 | 1.63 | 1.98 | 2.02 |
|  |  |  |  | (1.20, 3.26) | (1.22, 3.35) |
| **2011** | 6 (11.1) | 48 | 1.76 | 2.14 | 2.18 |
|  |  |  |  | (1.31, 3.49) | (1.33, 3.56) |
| **2012** | <5 (12.5) | 28 | 0.97 | 1.18 | 1.18 |
|  |  |  |  | (0.69, 2.04) | (0.69, 2.04) |
| **2013** | <5 (11.1) | 24 | 0.86 | 1.00 (Reference) | 1.00 (Reference) |
| **2014** | 8 (25.0) | 24 | 0.84 | 0.98 | 0.97 |
|  |  |  |  | (0.55, 1.72) | (0.55, 1.71) |
| **2015** | <5 (10.8) | 33 | 1.14 | 1.34 | 1.35 |
|  |  |  |  | (0.79, 2.27) | (0.80, 2.28) |
| **2016** | 5 (15.2) | 28 | 0.95 | 1.12 | 1.12 |
|  |  |  |  | (0.65, 1.92) | (0.65, 1.92) |
| **2017** | 5 (15.6) | 27 | 0.93 | 1.13 | 1.21 |
|  |  |  |  | (0.65, 1.95) | (0.70, 2.11) |
| **2018** | 10 (26.3) | 28 | 0.99 | 1.20 | 1.42 |
|  |  |  |  | (0.7, 2.08) | (0.81, 2.48) |
| **2019** | <5 (10.3) | 26 | 0.96 | 1.12 | 1.32 |
|  |  |  |  | (0.65, 1.96) | (0.75, 2.33) |

^a^ n=63 deaths excluded due to having congenital malformations, deformations and chromosomal abnormalities coded as cause of death in the cause of death unit record file.
